# Genomic Profiling of the Steroidogenic Acute Regulatory Protein in Breast Cancer: In Silico Assessments and a Mechanistic Perspective

**DOI:** 10.3390/cancers11050623

**Published:** 2019-05-04

**Authors:** Pulak R. Manna, Ahsen U. Ahmed, Shengping Yang, Madhusudhanan Narasimhan, Joëlle Cohen-Tannoudji, Andrzej T. Slominski, Kevin Pruitt

**Affiliations:** 1 Departments of Immunology and Molecular Microbiology, School of Medicine, Texas Tech University Health Sciences Center, Lubbock, TX 79430, USA; ahsen.ahmed@ttuhsc.edu (A.U.A.); kevin.pruitt@ttuhsc.edu (K.P.); 2 Internal Medicine, School of Medicine, Texas Tech University Health Sciences Center, Lubbock, TX 79430, USA; shengping.yang@ttuhsc.edu; 3 Pharmacology and Neuroscience, School of Medicine, Texas Tech University Health Sciences Center, Lubbock, TX 79430, USA; madhu.narasimhan@ttuhsc.edu; 4 Physiologie de l’axe gonadotrope U1133, Institut National de la Santé et de la Recherche Médicale, CNRS, Biologie Fonctionnelle et Adaptative UMR 8251, Université Paris Diderot, 75205 Paris, France; tannoudji@univ-paris-diderot.fr; 5 Department of Dermatology and Laboratory Medicine, Comprehensive Cancer Center, Cancer Chemoprevention Program, University of Alabama at Birmingham, Birmingham, AL 35294, USA; aslominski@uabmc.edu; 6 Veterans Administration Medical Center, Birmingham, AL 35294, USA

**Keywords:** hormone sensitive cancers, breast cancer, StAR, estrogen, steroidogenic enzymes

## Abstract

Cancer is a multifactorial condition with aberrant growth of cells. A substantial number of cancers, breast in particular, are hormone sensitive and evolve due to malfunction in the steroidogenic machinery. Breast cancer, one of the most prevalent form of cancers in women, is primarily stimulated by estrogens. Steroid hormones are made from cholesterol, and regulation of steroid/estrogen biosynthesis is essentially influenced by the steroidogenic acute regulatory (StAR) protein. Although the impact of StAR in breast cancer remains a mystery, we recently reported that StAR protein is abundantly expressed in hormone sensitive breast cancer, but not in its non-cancerous counterpart. Herein, we analyzed genomic profiles, hormone receptor expression, mutation, and survival for StAR and steroidogenic enzyme genes in a variety of hormone sensitive cancers. These profiles were specifically assessed in breast cancer, exploiting The Cancer Genome Atlas (TCGA) datasets. Whereas StAR and key steroidogenic enzyme genes evaluated (*CYP11A1, HSD3B*, *CYP17A1*, *CYP19A1*, and *HSD17B*) were altered to varying levels in these hormone responsive cancers, amplification of the *StAR* gene was correlated with poor overall survival of patients afflicted with breast cancer. Amplification of the *StAR* gene and its correlation to survival was also verified in a number of breast cancer studies. Additionally, TCGA breast cancer tumors associated with aberrant high expression of *StAR* mRNA were found to be an unfavorable risk factor for survival of patients with breast cancer. Further analyses of tumors, nodal status, and metastases of breast cancer tumors expressing *StAR* mRNA displayed cancer deaths in stage specific manners. The majority of these tumors were found to express estrogen and progesterone receptors, signifying a link between StAR and luminal subtype breast cancer. Collectively, analyses of genomic and molecular profiles of key steroidogenic factors provide novel insights that StAR plays an important role in the biologic behavior and/or pathogenesis of hormone sensitive breast cancer.

## 1. Introduction

The rate-limiting step in the regulation of steroid hormone biosynthesis is the transport of the substrate of all steroid hormones, cholesterol, from the outer to the inner mitochondrial membrane, a process that is predominantly mediated by the steroidogenic acute regulatory (StAR; also called STARD1) protein [1,2,3,4]. There is wealth of information that regulation of steroid biosynthesis is mediated by mechanisms that enhance the transcription, translation, or activity of StAR [2,4,5,6]. Noteworthy, whereas phosphorylation of StAR is associated with the optimal cholesterol transferring ability of the StAR protein in steroid biosynthesis, mutations in the *StAR* gene results in a protein that is nonfunctional and inactive in transporting cholesterol. In almost every system studied, agents/factors that influence StAR expression also influence steroid biosynthesis through endocrine, autocrine, and paracrine regulation in a variety of classical and non-classical steroidogenic tissues [2,4,7,8,9,10,11]. Following the transport of cholesterol, by StAR, to the inner mitochondrial membrane, the P450 side chain cleavage (P450scc) enzyme, encoded by the *CYP11A1* gene, catalyzes the first enzymatic step in steroidogenesis i.e., the conversion of cholesterol to pregnenolone [4,6]. In addition, CYP11A1 converts 7-dehydrocholesterol to 7-dehydropregneolone and activates vitamin D, emphasizing the importance of StAR to transport other substrates for non-canonical activity of CYP11A1 [12,13]. The first steroid, pregnenolone, is then metabolized to various sex steroids by a series of enzymes in target tissues. These enzymes include 3β-hydroxysteroid dehydrogenase (3β-HSD), 17α-monooxygenase, 17α-hydroxylase, 17,20-lyase (P45017α), aromatase, and 17β-HSD, which are encoded by the *HSD3B*, *CYP17A1*, *CYP19A1*, and *HSD17B* genes, respectively [4,8].

Steroid hormones are synthesized not only in endocrine tissues, but also in a variety of extra-gonadal/adrenal tissues, and they play crucial roles in diverse processes, ranging from development to homeostasis to carcinogenesis [4,10,11,14,15,16]. Of note, StAR mediates steroid biosynthesis by controlling the transport of cholesterol and, thus, its entry to the mitochondrial inner membrane is a key event in influencing various cholesterol/steroid led functions. Conversely, inappropriate regulation of StAR, involving cholesterol transport, might influence hormone dependent disorders. Accordingly, cholesterol and its metabolites have been shown to be involved in the etiology of a number of cancers [17,18]. Moreover, dysregulation of androgen and estrogen biosynthesis has long been implicated in the pathogenesis a variety of hormone sensitive cancers [16,19]. 

One of the most common malignancies in women is breast cancer, which is activated by estrogens, especially 17β-estradiol (E2), and it accounts for over one-fourth of all cancer cases [16,20,21,22]. The American Cancer Society estimated that 266,120 women were expected to be diagnosed with invasive breast cancer, with 40,920 deaths in 2018. Breast cancers are classified into four subtypes, i.e., luminal A, luminal B, HER2/ErbB2+ (human epidermal growth factor receptor 2/the erythroblastosis oncogene-B2 positive), and TNBC (triple negative breast cancer), based on estrogen receptor (ER), progesterone receptor (PR), and HER2 expression [23]. Hormone sensitive breast cancers predominantly express ER, especially ERα, and/or PR, and account for ~80% of all breast cancer cases. The remaining 15–20% cancers include HER2+ that expresses HER2, and TNBC that does not express ER, PR, and HER2 [24,25]. In this connection, it is worth noting that expression of the StAR protein has been shown to be markedly high in ER+/PR+ breast cancer, modest in TNBC, but little to none in normal mammary epithelial cells [5]. Additionally, accumulation of E2 mirrored StAR protein expression in both noncancerous and cancerous breast cell lines, suggesting that StAR plays a key role in the development of ER+/PR+ breast cancer. To obtain more insight into the association of StAR in breast cancer, genomic profiling of StAR and key steroidogenic enzyme genes were analyzed by exploiting two publicly available research databases: The Cancer Genome Atlas (TCGA, provisional for different cancer types) and cBioPortal (for independent breast cancer studies).

## 2. Materials and Methods

### 2.1. TCGA Hormone Responsive Cancer Tumors and Their Correlation to Copy Number Alterations of StAR and Steroidogenic Enzyme Genes 

TCGA genomic research databases were assessed for the following hormone sensitive cancers: breast (1080 tumors), colorectal (616 tumors), melanoma (367 tumors), ovarian (579 tumors), pancreatic (184 tumors), prostate (492 tumors), and uterine endometrial (539 tumors) [26,27,28,29]. These tumors were analyzed for DNA copy number alterations (CNAs) for StAR and key steroidogenic enzyme genes using the GISTIC 2.0. algorithm. CNA data were categorized as high-level amplification (+2 copies), gain (+1 copy), diploid (normal/no change), homozygous deletion (−2 copies), and hemizygous deletion (−1 copy). These analyses were performed using UCSC Xena [30] and/or cBioPortal Cancer Genomics [31,32] platforms. StAR CNA data were further evaluated for their correlation to StAR mRNA expression with RNA-Seq data, using the RSEM algorithm [33]. The correlation between StAR CNA and StAR mRNA levels was verified by Spearman’s rank coefficient analysis.

### 2.2. Expression of ER, PR, and HER2 in Breast Cancer Tumors

The predictive immunohistochemical (IHC) markers, employed in clinical settings to classify breast cancer tumors into biologically distinct subtypes with unique pathogenesis, were examined. The use of IHC to assess ER, PR, and HER2 expression status in breast cancer has been routinely performed in clinics. IHC based tumor classification was analyzed for ER, PR, and HER2 expression using TCGA breast cancer datasets. These receptors were also evaluated in a number of breast cancer publications and/or projects that are available in cBioPortal website [31,32]. 

### 2.3. Amplification of the StAR Gene in Breast Cancer Studies 

Amplification of the *StAR* gene was assessed in a variety of breast cancer publications/projects with cBioPortal browser. In particular, *StAR* gene amplification was analyzed in the following breast cancer studies: METABRIC (Molecular Taxonomy of Breast Cancer International Consortium), *Nature Communication* [34], (2173 tumors); breast cancer patient xenografts [35], (29 tumors); breast invasive carcinoma [36], (TCGA Cell 2015, 816 tumors); breast invasive carcinoma, [27], (TCGA Provisional; *Nature* 2012, 1080 tumors); metastatic breast cancer, *PLoS Medicine* [37], (216 tumors); and metastatic breast cancer (MBC) project (TCGA 2017, 103 tumors). These studies include mixed tumor types with variable numbers, in which amplification of the *StAR* gene and its correlation to overall survival, were evaluated, using available datasets. 

### 2.4. Mutational Portraits of the StAR Gene in TCGA Hormone Responsive Cancers

Mutation in the *StAR* gene was examined in different hormone responsive cancers by analyzing exome sequencing, utilizing TCGA datasets. Mutational analyses were limited for functional forms. Intronic, silent, or other forms of mutations were not considered. These analyses were performed using UCSC Xena platform [30]. Gene mutation frequency is described as a percentage of total number of tumors.

### 2.5. Expression of StAR mRNA in TCGA Breast Cancer Tumors and Their Correlation to TNM Stages

Expression of StAR mRNA, evaluated from RNA-Seq data, available for breast cancer tumors, was downloaded from TCGA and UCSC Xena websites. StAR mRNA expressed as upper quartile-normalized fragments per kilobase of transcript per million mapped reads (fpkm+uq+1), generated by TCGA, was plotted using the Box and Whisker plot [38]. The Box and Whisker plot depicts normal distribution of StAR mRNA and determines the median and quartiles in a statistical population.

The T (tumor), N (node), and M (metastasis) staging, is a globally recognized system for defining the extent of stage and/or spread of solid tumors for prognosis and treatment [39,40]. The TNM staging of TCGA breast cancer tumors, expressing StAR mRNA, was performed using the American Joint Committee on cancer classifications [39,40]. StAR mRNA/RNA-Seq data analyzed for various purposes are provided as an Excel file under Supplemental Materials. 

### 2.6. Generation of Kaplan-Meier Curves and Overall Survival Analyses

Kaplan-Meier curve is frequently used to determine survival analysis for clinical outcomes such as recovery rates, probability of death, and disappearance of a tumor [41]. Utilizing TCGA and/or cBioportal breast cancer tumor CNA data, Kaplan-Meier survival curves were generated using with (high level amplification) and without (diploid) amplification [42,43,44] for *StAR, CYP11A1, HSD3B1, CYP17A1, CYP19A1, HSD17B1*, and *HSD17B2* genes. For StAR, survival curve was also generated with and without (all tumors excluding homozygous deletion) amplification. Both *HSD17B1* and *HSD17B2* gene isoforms evaluated were based on their association with breast cancer [45]. Additionally, Kaplan-Meier survival curves were generated by dividing tumors into non-overlapping upper and lower groups based on two reports, with StAR mRNA values up to 50th percentile as low and above 50th as high [46]; and up to 25th percentile as low and above 25th percentile as high [47,48].

### 2.7. Statistical Analysis

Statistical analyses were performed using GraphPad Prism software (GraphPad, San Diego, CA, USA). Data represented are the mean ± SEM and analyzed using one-way analysis of variance (ANOVA) followed by post-hoc test. Spearman’s rank coefficient analysis was performed to determine the correlation between StAR CNA and StAR mRNA levels. The analysis of overall survival between groups was performed by log-rank Mantel-Cox method. A *p*-value less than 0.05 was considered statistically significant.

## 3. Results

### 3.1. Assessment of StAR CNAs in Various Hormone Sensitive Cancers 

Gene amplification, comprising oncogene activation, is a fundamental event in tumor progression [42]. The hypothesis that estrogen and/or androgen sensitive cancers involve gain of function of StAR in the transport of cholesterol, and thereby influence hormone sensitive cancers, was examined. Utilizing TCGA datasets, StAR CNA data were analyzed in a variety of hormone dependent cancers (Table 1). Breast cancer CNA data for StAR demonstrated ~13% high level amplification (138 tumors), ~25% gain (268 tumors), ~38% diploid (406 tumors), ~23% hemizygous deletion (252 tumors), and ~1.5% homozygous deletion (16 tumors). Tumor numbers altered in each category are shown in parentheses. Analysis of colorectal cancer CNA data for StAR resulted in ~2.5%, ~30%, ~44%, ~23%, and ~8% high level amplification, gain, diploid, hemizygous deletion, and homozygous deletion, respectively. Whereas StAR CNA data were found to be altered at varying levels, high level amplification was observed at 4.4% in pancreatic cancer. Likewise, melanoma, ovarian, prostate, and uterine endometrial cancer CNA data for StAR displayed ~0.3%, ~3.5%, ~2.9%, and ~1.9% high level amplification in these malignant tumors, respectively (Table 1). These data are consistent with previous detection of StAR in peripheral tissues and malignant tumors [4,49]. Higher amplification of the *StAR* gene (~13%) was next evaluated for its impact on breast cancer. 

### 3.2. Expression of ER, PR and HER2 in TCGA Breast Cancer Tumors

**Figure 1:**
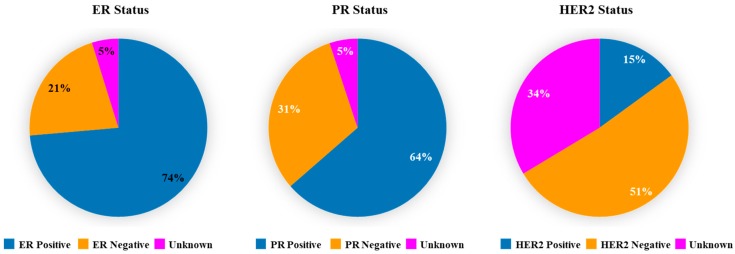
Expression of ER, PR and HER2 in The Cancer Genome Atlas (TCGA) breast cancer tumors. These tumors were previously stained with specific IHC markers in a clinical setting to classify into biologically distinct subtypes. Pie charts illustrate ER, PR, and HER2 expression in breast cancer tumors, which are presented as percentage of total numbers. Expression of these receptors was categorized as positive, negative, and unknown. The unknown category includes tumors in which IHC analysis was either not done or indeterminate or equivocal or data was not available.

To assess breast cancer subtype(s) in TCGA tumor datasets, expression of ER, PR and HER2 was examined. IHC data revealed differential expression of ER (74% positive, 21% negative, 5% unknown), PR (64% positive, 31% negative, 5% unknown), and HER2 (15% positive, 51% negative, 34% unknown) (Figure 1). These results indicate that TCGA breast cancer tumors are mostly ER+/PR+, representing they are largely luminal subtypes. 

### 3.3. Amplification of the StAR Gene in Breast Cancer and Its Correlation to Overall Survival

**Figure 2:**
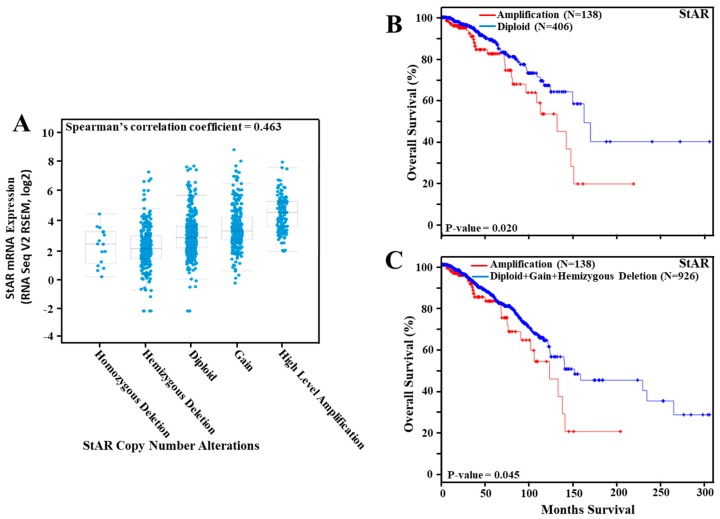
Frequency of StAR CNA data in breast cancer tumors and its correlation to overall survival. StAR CNA data were obtained from TCGA breast cancer tumor datasets with 1080 tumors. The CNA level was categorized as homozygous deletion, hemizygous deletion, diploid, gain, and high level amplification (**A**), utilizing cBioPortal browser, as described under Section 2. Breast cancer RNA-Seq data were assessed for StAR mRNA expression that positively correlated with StAR CNA data in different categories (**A**), which were presented in Y-axis and X-axis, respectively. Amplification of the *StAR* gene was evaluated for overall breast cancer survival (**B**,**C**). Kaplan-Meier curve was generated with TCGA breast cancer tumor CNA data, using with amplification (138 tumors) vs. without amplification (diploid, 406 tumors; **B**), or with a category (926 tumors; **C**) excluding homozygous deletion (16 tumors) of the *StAR* gene. Red and blue lines in panels B and C represent with and without amplification of the *StAR* gene, respectively.

Utilizing TCGA breast cancer data cohort, amplification of the *StAR* gene was examined for cancer survival. As illustrated in Figure 2A, StAR CNA data in different categories were positively correlated with StAR mRNA expression (RNA-Seq data). The correlation between StAR CNA and StAR mRNA levels was verified with Spearman’s correlation coefficient, i.e., 0.463. The analysis of Kaplan-Meier curve demonstrated that amplification of the *StAR* gene (~13%) was correlated with poor survival of breast cancer patients (*p*-value = 0.020). The median survival rate was noticeably reduced with amplification of the *StAR* gene when compared without amplification (Figure 2B). Similarly, the survival of breast cancer was affected (*p*-value = 0.045) when Kaplan-Meier curve was generated with and without (in which all tumors, excluding homozygous deletion, was included) *StAR* gene amplification (Figure 2C). 

### 3.4. StAR Gene Amplification, Hormone Receptor Expression, and Their Correlation to Cancer Survival in a Number of Breast Cancer Studies

**Figure 3:**
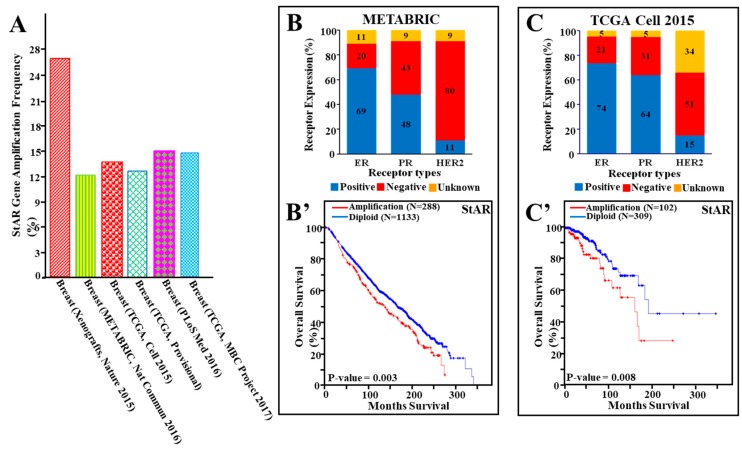
Amplification of the *StAR* gene, expression of hormone receptors, and their correlation to breast cancer survival in a number of publications/projects. Amplification of the *StAR* gene in different breast cancer studies, as available in cBioPortal, was analyzed. (**A**), amplification of the S*tAR* gene was evaluated in the following breast cancer studies: breast cancer patient xenografts, Nature 2015 (29 tumors), [35]; breast METABRIC, *Nature Communication* 2016 (2173 tumors) [34]; breast TCGA Cell 2015, (816 tumors), [36]: TCGA Provisional (1080 tumors), [27]; breast *PLoS Medicine* 2016 (216 tumors), [37]; and TCGA Metastatic Breast Cancer (MBC) Project 2017 (103 tumors). Receptor expression was categorized as positive, negative, and unknown, and presented as percentages of total number of tumors (**B**,**C**), as described in the legend of Figure 1. Levels of ER, PR, and HER2 expression and their correlation to overall survival were analyzed for METABRIC (**B**,**B’**) and TCGA Cell 2015 (**C**,**C’**) studies. Kaplan-Meier survival curves were generated with METABRIC (red line, 288 tumors; blue line, 1133 tumors) and *Cell* 2015 (red line, 102 tumors; blue line, 309 tumors) CNA data, using tumors with amplification and without amplification (diploid) of the *StAR* gene.

To better understand involvement of *StAR* gene amplification in breast cancer deaths, genomic data from a number of publications/projects, as available in cBioPortal, were analyzed. As depicted in Figure 3A, amplification of the *StAR* gene was observed between 12% and 26% in all breast cancer studies examined. Specifically, amplification of the *StAR* gene was 26% in a breast cancer patient xenografts study, 12% in breast cancer METABRIC, and 13% and 14% in two independent publications associated with breast invasive carcinomas, and 15% each in two independent metastatic breast cancer studies (specified in Section 2). 

In additional analyses, ER, PR, and HER2 expression, amplification of the *StAR* gene and its correlation to overall survival, were evaluated. In METABRIC study, breast cancer tumors (2173 tumors) were 69% ER+, 48% PR+, and 80% HER−, representing a mixed subtype, in which amplification of the *StAR* gene (~12%) affected the survival (*p*-value = 0.003) of breast cancer (Figure 3B,B’). In a breast invasive carcinoma study (Cell 2015, 816 tumors), amplification of the *StAR* gene (~14%), associated with 74% ER+, 64% PR+, and 51% HER2− (15% HER2+), was found to correlate (*p*-value = 0.008) with poor breast cancer survival (Figure 3C,C’). These data corroborate the findings presented in Figure 2B,C, and demonstrate that amplification of the *StAR* gene is correlated with poor survival of patients with luminal subtype breast cancer. Survival data were not available for other studies included in Figure 3A. 

### 3.5. Amplification of Steroidogenic Enzyme Genes and Their Correlation to Overall Breast Cancer Survival 

**Figure 4:**
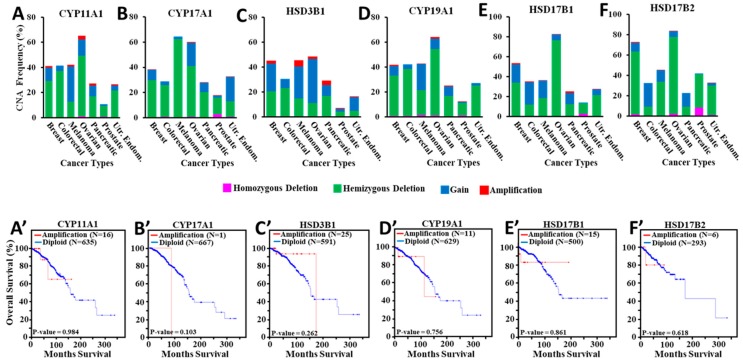
Analyses of CNA data for various steroidogenic enzyme genes in different hormone sensitive cancers and their correlation to breast cancer survival. TCGA CNA data analyzed for different cancers were the following: breast cancer tumors (1080 cases), colorectal (616 cases), melanoma (367 cases), ovarian (579 cases), pancreatic (184 cases), prostate (492 cases), and uterine endometrial (539 cases). Bar graphs illustrate CNA data for steroidogenic enzyme genes: *CYP11A1* (**A**), *CYP17A1* (**B**), *HSD3B1* (**C**), *CYP19A1* (**D**), *HSD17B1* (**E**), and *HSD17B2* (**F**). Amplification of these genes was analyzed for overall breast cancer survival (**A’**–**F’**). Kaplan-Meier survival curves were generated with and without (diploid) amplification of the following steroidogenic enzyme genes: *CYP11A1* (**A’**; red line, 16 tumors; blue line, 635 tumors), *CYP17A1* (**B’**; red line, 1 tumor; blue line, 667 tumors), *HSD3B1* (**C’**; red line, 25 tumors; blue line, 591 tumors), *CYP19A1* (**D’**; red line, 11 tumors; blue line, 629 tumors), *HSD17B1* (**E’**; red line, 15 tumors; blue line, 500 tumors), and *HSD17B2* (**F’**; red line, 6 tumors; blue line, 293 tumors). Red and blue lines represent with and without amplification of target genes, respectively. Utr. Endom., Uterine Endometrial.

Estrogen plays an important role in stimulating breast cancer. The involvement of key steroidogenic enzyme genes (Supplementary Figure S1) to estrogen synthesis was next evaluated for their association to breast cancer survival utilizing TCGA data cohort. The data presented in Figure 4A–F illustrate bar graphs of different CNA frequencies (high level amplification (red), gain (blue), hemizygous deletion (green), and homozygous deletion (pink) for *CYP11A1*, *CYP17A1*, *HSD3B1, CYP19A1, HSD17B1,* and *HSD17B2* enzyme genes in different hormone sensitive cancers. Diploid category is not shown in these bar diagrams for easier visualization. CNA data demonstrate that the *CYP11A1* gene was amplified at ~1.5%, ~1.4%, ~3%, ~1.6%, and ~1.1% in breast, melanoma, ovarian, pancreatic, and uterine endometrial cancers, respectively (Figure 4A). No amplification of the *CYP11A1* gene was observed in prostate and colorectal cancers. The *CYP17A1* gene was amplified less than 1% in all cancer types analyzed (Figure 4B). Amplification of the *HSD3B1* gene was highest (~5%) in melanoma and none in colorectal cancer (Figure 4C). In breast cancer, this gene was amplified at ~2.3%. Amplification of the *CYP19A1* gene (aromatase) was ~1%, ~0.5%, ~0.3%, ~0.2%, ~1.6%, 0%, and ~0.2% in breast, colorectal, melanoma, ovarian, pancreatic, prostate, and uterine endometrial cancers, respectively (Figure 4D). Additionally, both *HSD17B1* and *HSD17B2* gene isoforms were found to be amplified minimally (0–1.4%) in different hormone sensitive cancers studied (Figure 4E,F). These *HSD17B1* and *HSD17B2* isoforms were amplified at ~1.4% and ~0.6% in breast cancer, respectively. These results are in support of previous studies that demonstrated upregulation of aberrant steroidogenesis during tumor progression [49,50].

The amplification of these steroidogenic enzyme genes in breast cancer survival was next evaluated. As determined by Kaplan-Meier survival analyses, amplification of the *CYP11A1* gene was not associated (*p*-value = 0.984) with breast cancer survival (Figure 4A’). Similarly, both *CYP17A1* and *HSD3B1* gene amplifications were not found to affect the survival of breast cancer, in which *p*-values were 0.103 and 0.262, respectively (Figure 4B’,C’). Kaplan-Meier survival analysis revealed that amplification of the *CYP19A1* gene was not correlated (*p*-value = 0.756) with breast cancer survival (Figure 4D’). Additionally, amplification of both *HSD17B1* and *HSD17B2* gene isoforms did not affect the survival of breast cancer, where *p*-values were 0.861 and 0.618, respectively (Figure 4E’,F’). These data indicate that none of these steroidogenic enzyme genes were either substantially amplified or affected the survival of ER+/PR+ breast cancer.

### 3.6. Assessment of StAR Gene Mutation in Hormone Sensitive Cancers

TCGA hormone responsive cancer datasets were analyzed for identifying mutation(s) in the *StAR* gene, which has been shown to affect the biological activity of the StAR protein in steroid biosynthesis [4,51]. As determined by exome sequencing, no mutations in the *StAR* gene were observed in breast (982 tumors) and prostate (499 tumors) cancers, suggesting StAR is functionally active in mobilizing cholesterol to the mitochondria. However, one mutation in the *StAR* gene was identified in each of the following cancers: colorectal (one out of 223 tumors; 0.45%), pancreatic (one out of 150 tumors; 0.67%), and ovarian (one out of 316 tumors; 0.32%). In melanoma and uterine endometrial carcinomas, five (368 tumors; 1.36%) and four (248 tumors; 1.61%) mutations were observed in the *StAR* gene, respectively (Supplementary Figure S2). The absence of mutation in the *StAR* gene, especially in breast cancer, suggests that amplification of the *StAR* gene is culpable in the transport of excess cholesterol to the inner mitochondrial membrane, resulting in increased estrogen synthesis which would promote tumorigenesis. 

### 3.7. Expression of StAR mRNA in TCGA Breast Cancer Tumors and Its Association to Overall Survival

**Figure 5:**
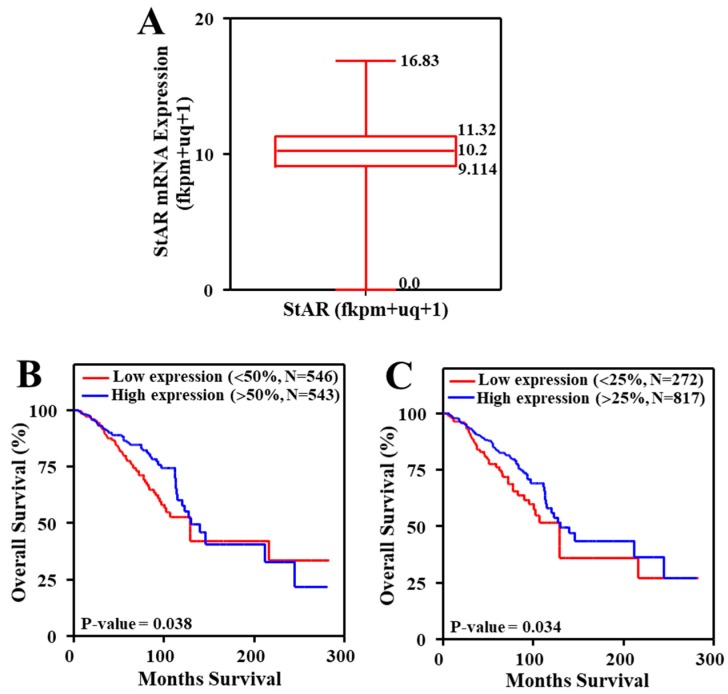
Expression of StAR mRNA in TCGA breast cancer tumors using the Box and Whisker Plot, and generation of Kaplan-Meier curves with low vs. high StAR levels. StAR mRNA expression was illustrated as fkpm+uq+1, generated by TCGA (1089 tumors), and visualized with the Box and Whisker Plot (**A**), as described in Section 2. Shown are 25th (9.114) and 75th (11.32) percentiles with a median of 10.2 (indicated by a horizontal line). Kaplan-Meier survival curves were generated with StAR mRNA values up to 50th percentile (<10.2; 546 tumors) as low and above 50th percentile (>10.2; 543 tumors) as high (**B**), and up to 25th percentile as low (<9.114; 272 tumors) and above 25th percentile (>9.114; 817 tumors) as high (**C**). Red and blue lines represent low and high StAR mRNA expression, respectively.

TCGA breast cancer tumor datasets were assessed for StAR mRNA expression. As illustrated by the Box and Whisker plot, StAR mRNA expression was represented as fkpm+uq+1 (obtained from RNA-Seq data), in which normal distribution across the population was visualized as 25th (9.114) and 75th (11.32) percentiles with a median of 10.2 (Figure 5A).

To better understand the involvement of StAR in breast cancer, TCGA breast cancer tumors expressing StAR mRNA were verified for survival analyses with two different quartile combinations. As depicted in Figure 5B, Kaplan-Meier curve generated with StAR mRNA values up to 50th percentile (<10.2) as low and above 50th percentile (>10.2) as high [46], was found to correlate with poor survival (*p*-value = 0.038) of patients with breast cancer. In a different category, StAR mRNA values up to 25th percentile (<9.114) as low and above 25th percentile (>9.114) as high [48], showed qualitatively similar effect (*p* = 0.034) on the survival of breast cancer (Figure 5C). These data suggest that higher expression of StAR mRNA can be a risk factor for poor survival of patients with breast cancer. 

### 3.8. TNM Staging and Its Correlation to Breast Cancer Deaths

To obtain more insight in to the impact of StAR in breast cancer deaths, TCGA breast cancer tumors expressing StAR mRNA were analyzed in conjunction with the TNM staging. Specifically, different TNM stages were evaluated with low and high StAR mRNA levels with two quantile combinations as those utilized in Figure 5B,C. The results presented in Table 2 demonstrate TNM stage specific effects of tumors and their correlation to breast cancer deaths. These results show that breast cancer deaths were found to be coordinately associated with not only to increased tumor sizes, but also to lymph nodes in stage dependent manners. Additionally, tumor metastasis (M1) markedly affected the survival of breast cancer when compared with no metastasis (M0) in both low and high categories (Table 2). Specifically, the results obtained with TNM stages confirm the Kaplan-Meier survival data presented in Figure 5B,C. Altogether, genomic analyses of key steroidogenic factors, within the context of TCGA breast cancer datasets, indicated that aberrant amplification/ expression of the *StAR* gene is involved, at least in part, in poor survival of ER+/PR+ breast cancer patients. These results are in support of our recent finding that demonstrated that StAR protein is abundantly expressed in hormone sensitive breast cancer [5]. 

## 4. Discussion 

Abnormality in gene expression is responsible for anomalous growth of cells connecting tumor progression. The majority of the human genome is transcribed, but not translated, and gene amplification, involving oncogene activation, is a fundamental event in cancers. Hormone responsive cancers, especially breast cancer, are most common globally. Since StAR plays an indispensable role in the regulation of steroidogenesis, its expression must be finely regulated to appropriate functioning of steroid led activities. Conversely, dysregulation of steroid biosynthesis has been implicated in the pathophysiology of a number of relevant cancers. While StAR’s involvement in breast malignancy remains obscure, we recently reported that both StAR protein expression and E2 synthesis are profoundly higher in ER+/PR+ breast cancer cell lines, when compared their levels with either non-cancerous mammary epithelial cells or TNBC [5]. By analyzing genomic profiles of StAR and steroidogenic enzyme genes for several hormone sensitive cancers, our data extend previous observations and provide novel insight that aberrant high amplification/expression of the *StAR* gene is correlated with poor survival of patients with breast cancer. 

The comprehensive analyses of TCGA and cBioPortal research datasets for various hormone responsive cancers demonstrate that *StAR* gene is amplified (associated with a positive correlation between StAR CNA and StAR mRNA levels), but not mutated, in luminal subtype breast cancer. Specifically, the association of StAR with ER+/PR+ breast cancer indicates that StAR acts as a tumor promoter in the most prevalent hormone sensitive breast cancer. Several lines of evidence demonstrate a close correlation between StAR mRNA and StAR protein synthesis which parallels the synthesis of steroids in a variety of target tissues [4,7,10,52]. The involvement of StAR in breast cancer appeared specific, as translocator protein (TSPO), a mitochondrial factor involved in steroidogenesis [53,54], was not connected (*TSPO* gene was amplified at 0.7% with a *p*-value = 0.540) with cancer deaths (data not illustrated). The mechanism accounting for estrogen sensitive ovarian and endometrial cancers, connecting mutations in the *StAR* gene, remains unclear, and may involve one or more compensatory event(s), including involvement of StAR related lipid transfer proteins 3-6 (STARD3-6) and/or other factors involved in cholesterol trafficking [55,56]. Of note, the late endosomal membrane protein STARD3 (also known as metastatic lymph node 64), with ~37% C-terminal homology to StAR, was initially cloned as a gene amplified in the breast, gastric, and esophageal cancers [57,58]. It has previously been shown that overexpression of STARD3 is associated with increased cholesterol biosynthesis in HER2+ breast cancer subtype [59,60]. Regardless of the influence of these transporters, cholesterol and its oxygenated derivatives were demonstrated to be involved in the pathophysiology of a number of hormone sensitive malignancies, including breast cancer [17,18]. Studies have also reported that both cholesterol and its metabolites, including 27-hydroxycholesterol (27-HC) and 6-oxocholestan-diol, are capable to accelerate and/or enhance breast tumorigenesis [17,61,62]. Noteworthy, 27-HC is a ligand for ER and liver X receptor (LXR), in which the effects of 27-HC on tumor formation and growth are dependent on ER, while the action of this oxysterol involves LXR in tumor metastasis in mouse breast cancer models [17]. Whereas an overwhelming amount of evidence indicates the involvement of cholesterol in hormone sensitive breast cancer, epidemiologic findings are contradictory, requiring future studies to assess whether total cholesterol and its metabolites, high-density lipoprotein, or low-density lipoprotein influence cancer development and progression.

Almost all proteins in eukaryotic cells are modified by various post-translational modifications (PTMs) that influence protein function. We recently identified that StAR is a novel acetylated protein in ER+ breast cancer cells, in which three acetyl lysine residues were recognized endogenously, surmising they contribute to higher accumulation E2 in these cells [5]. It is plausible that both higher expression and activity of StAR facilitate abnormal cholesterol delivery to the mitochondrial inner membrane and, as a consequence, precursor availability for estrogen in promoting breast tumorigenesis. This reinforces the notion that estrogen levels in the majority of hormone sensitive malignant breast tumors can be 10–30 times higher than those found in either circulation or non-cancerous counterparts [16,21,63,64]. Previously, we [14,65,66] and others [67,68] have reported that cAMP mediated mechanisms phosphorylate StAR and this PTM enhances the optimal cholesterol transferring ability of the StAR protein in steroid biosynthesis. Despite the regulatory events involved, the impact of StAR to serve as a risk factor in affecting the survival of ER+/PR+ breast cancer opens up a new avenue in breast cancer research. 

A notable aspect of the present findings is that amplification of the *CYP19A1* gene (aromatase), within the context of TCGA data cohort, was not correlated with breast cancer death [16], even though aromatase is the rate-limiting enzyme in estrogen biosynthesis. Expression of aromatase has been shown to be high in both non-cancerous and cancerous breast cell lines, suggesting its relevance in a number of physiological and pathophysiological events [5,69]. There is increasing evidence that enhanced expression/activity of aromatase is one of the key events for elevated intra-tumoral production of estrogen in malignant breast tissues [16,21,70,71]. Estrogen is also produced by the action of the 17β-HSD enzyme, and CNA data revealed that the *HSD17B* gene was neither significantly amplified nor connected with the survival of hormone sensitive breast cancer. These data imply that StAR mediated delivery of excess cholesterol, resulting in a substantial increase in estrogen accumulation, appears to be a fundamental event in the development of hormone sensitive breast cancer. In accordance with this, preliminary data obtained reveal that the expression of both StAR mRNA and StAR protein was markedly high in transgenic (Tg) mouse models of breast cancer, activated by MMTV promoter driven cNeu and H-Ras oncogenes, and polyomavirus, in comparison to nearly undetectable level of StAR in normal Tg mammary tissue. 

Estrogen is primarily produced in the ovaries via the classical steroidogenic pathway through the synthesis of androstenedione and testosterone from cholesterol (in which StAR plays a permissive role) in the theca cells. These androgens are then converted to estrogens in granulosa cells. In peri- and post-menopausal women, extra ovarian tissues become a major source for estrogen synthesis [72]. This transition is critical since most hormone sensitive cancers, including breast, occur over the age of 50, in which estrogens synthesized in peripheral tissues are believed to play pivotal roles [63,64]. The plasma androgen level in post-menopausal women, with the loss of ovarian estrogen production, remains stable for years. Utilizing the non-classical pathway, these androgens are converted to estrogens in peripheral tissues. In addition to peripheral estrogen that reaches the tumor site through systemic circulation, estrogen is also synthesized locally in malignant breast tumors [16,21,63,64]. Breast cancer tumors in TCGA datasets were predominantly ER+/PR+, in which aberrant high expression of *StAR* mRNA, was found to affect poor survival of breast cancer. Further analyses of these tumors, expressing StAR mRNA, demonstrated increasing patterns of breast cancer deaths with advanced TNM stages. It should be noted, however, while breast cancer deaths were steadily increased with various TNM stages, they were not coordinately associated with StAR mRNA expression, which could be due to tumor numbers, tumor stages, or involvement of additional factors. 

## 5. Conclusions

Analyses of molecular genomic profiling of steroidogenic factors associated with TCGA and cBioPortal research datasets revealed that abundant amplification and/or expression of the StAR gene is connected with poor survival of patients with luminal subtype breast cancer. This is in support of our recent report that demonstrated that StAR protein, concomitant with E2 synthesis, is markedly expressed in ER+/PR+ breast cancer, in comparison to nearly undetectable to modest StAR and E2 levels in non-cancerous mammary epithelial cells [5]. Based on these data (albeit limited), it is highly likely that StAR facilitates abnormal delivery of cholesterol to the inner mitochondria, resulting in adequate availability of precursors for E2 overproduction, which could be a plausible mechanism in the development and growth of hormone sensitive breast cancer. Furthermore, the results of in silico analyses, together with our in vitro data reported recently, attest that StAR can serve as a novel prognostic marker in ER+/PR+ breast cancer, whereas its inhibition, involving E2 synthesis, by a number of histone deacetylase inhibitors, might have therapeutic implications in the prevention/treatment of this devastating disease. The present data indicating the involvement of the classical pathway in intra-tumoral androgen/estrogen synthesis points to an additional new mechanism in growth and development of ER+/PR+ breast and/or other pertinent cancers, even though overexpression of aromatase, resulting in an increase in estrogen synthesis through the non-classical pathway is well established. Whereas *StAR* gene is highly amplified/expressed in hormone sensitive breast cancer, its association with HER2 and TNBC subtypes remains to be elucidated.

## Figures and Tables

**Table 1:** DNA copy number alterations of the steroidogenic acute regulatory (*StAR*) gene in different hormone responsive cancers.

CNAs	Breast *N* (%)	Colorectal *N* (%)	Melanoma *N* (%)	Ovarian *N* (%)	Pancreatic *N* (%)	Prostate *N* (%)	Uterine Endometrial *N* (%)
Homozygous Deletion	16 (1.48)	8 (1.30)	3 (0.82)	7 (1.21)	0 (0.00)	32 (6.50)	7 (1.30)
Hemizygous Deletion	252 (23.33)	140 (22.73)	69 (18.80)	215 (37.13)	42 (22.83)	133 (27.03)	66 (12.24)
Diploid	406 (37.59)	271 (43.99)	184 (50.14)	220 (38.00)	109 (59.24)	258 (52.44)	345 (64.01)
Gain	268 (24.81)	182 (29.55)	110 (29.97)	117 (20.21)	25 (13.59)	55 (11.18)	111 (20.59)
High Level Amplification	138 (12.78)	15 (2.44)	1 (0.27)	20 (3.45)	8 (4.35)	14 (2.85)	10 (1.86)
Total Number of Tumors	1080	616	367	579	184	492	539

StAR CNA data were assessed for the following cancer tumors: breast (1080 cases), colorectal (616 cases), melanoma (367 cases), ovarian (579 cases), pancreatic (184 cases), prostate (492 cases), and uterine endometrial (539 cases). The CNA level was categorized as homozygous deletion, hemizygous deletion, diploid, gain, and high level amplification, as described under Section 2. *N* (%) = number of tumors with percentages in parentheses.

**Table 2:** T, N, M staging of TCGA breast cancer tumors segregated for low and high StAR mRNA expression based on two different quartile combinations, and their correlation to patient deaths.

TNM Staging	Low Expression (<50%)			High Expression (>50%)			Low Expression (<25%)			High Expression (>25%)		
	Tumor Nos.	Death Nos.	% Deaths	Tumor Nos.	Death Nos.	% Deaths	Tumor Nos.	Death Nos.	% Deaths	Tumor Nos.	Death Nos.	% Deaths
T1	120	17	14.2	158	16	10.1	54	8	14.8	224	25	11.2
T2	336	44	13.1	294	33	11.2	169	25	14.8	461	52	11.3
T3	63	11	17.5	75	14	18.7	32	9	28.1	106	16	15.1
T4	26	8	30.8	14	7	50	16	5	31.3	24	10	41.7
N0	257	22	8.6	257	22	8.6	120	15	12.5	394	29	7.4
N1	169	31	18.3	190	28	14.7	91	19	20.1	268	40	14.9
N2	75	16	21.3	45	6	13.3	38	6	15.8	82	16	19.5
N3	33	5	15.2	45	12	26.7	16	4	25.0	60	11	18.3
M0	459	62	13.5	621	32	5.2	224	26	11.6	672	83	12.4
M1	13	10	76.9	8	5	62.5	4	2	50	12	9	75.0

TCGA breast cancer tumors expressing low and high StAR mRNA levels (as specified) were categorized based on tumor sizes (T1–T4), nodal variations (N0–N3), and metastasis and non-metastasis (M0–M1), as described in Section 2.

## Supplementary Materials

The following are available online at https://www.mdpi.com/2072-6694/11/5/623/s1, Figure S1: Steroid biosynthetic pathway. Figure S2: Assessment of mutation in the StAR gene in a variety of hormone sensitive cancers. Supplementary Excel file: StAR mRNA (RNA-Seq) data for overall survival analysis.

## References

[1] Manna P.R., Stocco D.M. (2005). Regulation of the steroidogenic acute regulatory protein expression: Functional and physiological consequences. Curr. Drug Targets Immune Endocr. Metabol. Disord..

[2] Manna P.R., Dyson M.T., Stocco D.M. (2009). Regulation of the steroidogenic acute regulatory protein gene expression: Present and future perspectives. Mol. Hum. Reprod..

[3] Papadopoulos V., Miller W.L. (2012). Role of mitochondria in steroidogenesis. Best Pract. Res. Clin. Endocrinol. Metab..

[4] Manna P.R., Stetson C.L., Slominski A.T., Pruitt K. (2016). Role of the steroidogenic acute regulatory protein in health and disease. Endocrine.

[5] Manna P.R., Ahmed A.U., Vartak D., Molehin D., Pruitt K. (2019). Overexpression of the steroidogenic acute regulatory protein in breast cancer: Regulation by histone deacetylase inhibition. Biochem. Biophys. Res. Commun..

[6] Miller W.L., Bose H.S. (2011). Early steps in steroidogenesis: Intracellular cholesterol trafficking. J. Lipid Res..

[7] Manna P.R., Dyson M.T., Eubank D.W., Clark B.J., Lalli E., Sassone-Corsi P., Zeleznik A.J., Stocco D.M. (2002). Regulation of steroidogenesis and the steroidogenic acute regulatory protein by a member of the cAMP response-element binding protein family. Mol. Endocrinol..

[8] Miller W.L., Auchus R.J. (2011). The molecular biology, biochemistry, and physiology of human steroidogenesis and its disorders. Endocr. Rev..

[9] Manna P.R., Cohen-Tannoudji J., Counis R., Garner C.W., Huhtaniemi I., Kraemer F.B., Stocco D.M. (2013). Mechanisms of action of hormone sensitive lipase in mouse Leydig cells: Its role in the regulation of the steroidogenic acute regulatory protein. J. Biol. Chem..

[10] Manna P.R., Stetson C.L., Daugherty C., Shimizu I., Syapin P.J., Garrel G., Cohen-Tannoudji J., Huhtaniemi I., Slominski A.T., Pruitt K. (2015). Up-regulation of steroid biosynthesis by retinoid signaling: Implications for aging. Mech. Ageing. Dev..

[11] Slominski A.T., Manna P.R., Tuckey R.C. (2015). On the role of skin in the regulation of local and systemic steroidogenic activities. Steroids.

[12] Slominski A., Semak I., Zjawiony J., Wortsman J., Li W., Szczesniewski A., Tuckey R.C. (2005). The cytochrome P450scc system opens an alternate pathway of vitamin D3 metabolism. FEBS J..

[13] Slominski A.T., Li W., Kim T.K., Semak I., Wang J., Zjawiony J.K., Tuckey R.C. (2015). Novel activities of CYP11A1 and their potential physiological significance. J. Steroid Biochem. Mol. Biol..

[14] Manna P.R., Slominski A.T., King S.R., Stetson C.L., Stocco D.M. (2014). Synergistic Activation of Steroidogenic Acute Regulatory Protein Expression and Steroid Biosynthesis by Retinoids: Involvement of cAMP/PKA Signaling. Endocrinology.

[15] Slominski A.T., Manna P.R., Tuckey R.C. (2014). Cutaneous glucocorticosteroidogenesis: Securing local homeostasis and the skin integrity. Exp. Dermatol..

[16] Manna P.R., Molehin D., Ahmed A.U. (2016). Dysregulation of Aromatase in Breast, Endometrial, and Ovarian Cancers: An Overview of Therapeutic Strategies. Prog. Mol. Biol. Transl. Sci..

[17] Nelson E.R., Wardell S.E., Jasper J.S., Park S., Suchindran S., Howe M.K., Carver N.J., Pillai R.V., Sullivan P.M., Sondhi V. (2013). 27-Hydroxycholesterol links hypercholesterolemia and breast cancer pathophysiology. Science.

[18] Silvente-Poirot S., Dalenc F., Poirot M. (2018). The Effects of Cholesterol-Derived Oncometabolites on Nuclear Receptor Function in Cancer. Cancer Res..

[19] Brodie A., Njar V., Macedo L.F., Vasaitis T.S., Sabnis G. (2009). The Coffey Lecture: Steroidogenic enzyme inhibitors and hormone dependent cancer. Urol. Oncol..

[20] Richie R.C., Swanson J.O. (2003). Breast cancer: A review of the literature. J. Insur. Med..

[21] Bulun S.E., Lin Z., Zhao H., Lu M., Amin S., Reierstad S., Chen D. (2009). Regulation of aromatase expression in breast cancer tissue. Ann. N. Y. Acad. Sci..

[22] Folkerd E., Dowsett M. (2013). Sex hormones and breast cancer risk and prognosis. Breast.

[23] Cheang M.C., Martin M., Nielsen T.O., Prat A., Voduc D., Rodriguez-Lescure A., Ruiz A., Chia S., Shepherd L., Ruiz-Borrego M. (2015). Defining breast cancer intrinsic subtypes by quantitative receptor expression. Oncologist.

[24] Prat A., Pineda E., Adamo B., Galvan P., Fernandez A., Gaba L., Diez M., Viladot M., Arance A., Munoz M. (2015). Clinical implications of the intrinsic molecular subtypes of breast cancer. Breast.

[25] Dai X., Li T., Bai Z., Yang Y., Liu X., Zhan J., Shi B. (2015). Breast cancer intrinsic subtype classification, clinical use and future trends. Am. J. Cancer Res..

[26] Cancer Genome Atlas Research Network (2011). Integrated genomic analyses of ovarian carcinoma. Nature.

[27] Cancer Genome Atlas Network (2012). Comprehensive molecular portraits of human breast tumours. Nature.

[28] Cancer Genome Atlas N. (2012). Comprehensive molecular characterization of human colon and rectal cancer. Nature.

[29] Kandoth C., Schultz N., Cherniack A.D., Akbani R., Liu Y., Shen H., Robertson A.G., Pashtan I., Shen R., Benz C.C. (2013). Integrated genomic characterization of endometrial carcinoma. Nature.

[30] Casper J., Zweig A.S., Villarreal C., Tyner C., Speir M.L., Rosenbloom K.R., Raney B.J., Lee C.M., Lee B.T., Karolchik D. (2018). The UCSC Genome Browser database: 2018 update. Nucleic Acids Res..

[31] Cerami E., Gao J., Dogrusoz U., Gross B.E., Sumer S.O., Aksoy B.A., Jacobsen A., Byrne C.J., Heuer M.L., Larsson E. (2012). The cBio cancer genomics portal: An open platform for exploring multidimensional cancer genomics data. Cancer Discov..

[32] Gao J., Aksoy B.A., Dogrusoz U., Dresdner G., Gross B., Sumer S.O., Sun Y., Jacobsen A., Sinha R., Larsson E. (2013). Integrative analysis of complex cancer genomics and clinical profiles using the cBioPortal. Sci. Signal..

[33] Li B., Dewey C.N. (2011). RSEM: Accurate transcript quantification from RNA-Seq data with or without a reference genome. BMC Bioinform..

[34] Pereira B., Chin S.F., Rueda O.M., Vollan H.K., Provenzano E., Bardwell H.A., Pugh M., Jones L., Russell R., Sammut S.J. (2016). The somatic mutation profiles of 2,433 breast cancers refines their genomic and transcriptomic landscapes. Nat. Commun..

[35] Eirew P., Steif A., Khattra J., Ha G., Yap D., Farahani H., Gelmon K., Chia S., Mar C., Wan A. (2015). Dynamics of genomic clones in breast cancer patient xenografts at single-cell resolution. Nature.

[36] Ciriello G., Gatza M.L., Beck A.H., Wilkerson M.D., Rhie S.K., Pastore A., Zhang H., McLellan M., Yau C., Kandoth C. (2015). Comprehensive Molecular Portraits of Invasive Lobular Breast Cancer. Cell.

[37] Lefebvre C., Bachelot T., Filleron T., Pedrero M., Campone M., Soria J.C., Massard C., Levy C., Arnedos M., Lacroix-Triki M. (2016). Mutational Profile of Metastatic Breast Cancers: A Retrospective Analysis. PLoS Med..

[38] Bullard J.H., Purdom E., Hansen K.D., Dudoit S. (2010). Evaluation of statistical methods for normalization and differential expression in mRNA-Seq experiments. BMC Bioinform..

[39] O’Sullivan B., Brierley J., Byrd D., Bosman F., Kehoe S., Kossary C., Pineros M., Van Eycken E., Weir H.K., Gospodarowicz M. (2017). The TNM classification of malignant tumours-towards common understanding and reasonable expectations. Lancet Oncol..

[40] Lydiatt W.M., Patel S.G., O’Sullivan B., Brandwein M.S., Ridge J.A., Migliacci J.C., Loomis A.M., Shah J.P. (2017). Head and Neck cancers-major changes in the American Joint Committee on cancer eighth edition cancer staging manual. CA Cancer J. Clin..

[41] Sedgwick P. (2014). How to read a Kaplan-Meier survival plot. BMJ.

[42] Slamon D.J., Clark G.M., Wong S.G., Levin W.J., Ullrich A., McGuire W.L. (1987). Human breast cancer: Correlation of relapse and survival with amplification of the HER-2/neu oncogene. Science.

[43] Schneiderman J., London W.B., Brodeur G.M., Castleberry R.P., Look A.T., Cohn S.L. (2008). Clinical significance of MYCN amplification and ploidy in favorable-stage neuroblastoma: A report from the Children’s Oncology Group. J. Clin. Oncol..

[44] Liu L., Kimball S., Liu H., Holowatyj A., Yang Z.Q. (2015). Genetic alterations of histone lysine methyltransferases and their significance in breast cancer. Oncotarget.

[45] Hilborn E., Stal O., Jansson A. (2017). Estrogen and androgen-converting enzymes 17beta-hydroxysteroid dehydrogenase and their involvement in cancer: With a special focus on 17beta-hydroxysteroid dehydrogenase type 1, 2, and breast cancer. Oncotarget.

[46] Giovannetti E., Wang Q., Avan A., Funel N., Lagerweij T., Lee J.H., Caretti V., van der Velde A., Boggi U., Wang Y. (2014). Role of CYB5A in pancreatic cancer prognosis and autophagy modulation. J. Natl. Cancer Inst..

[47] Yoshihara K., Shahmoradgoli M., Martinez E., Vegesna R., Kim H., Torres-Garcia W., Trevino V., Shen H., Laird P.W., Levine D.A. (2013). Inferring tumour purity and stromal and immune cell admixture from expression data. Nat. Commun..

[48] Geng X., Liu Y., Diersch S., Kotzsch M., Grill S., Weichert W., Kiechle M., Magdolen V., Dorn J. (2017). Clinical relevance of kallikrein-related peptidase 9, 10, 11, and 15 mRNA expression in advanced high-grade serous ovarian cancer. PLoS ONE.

[49] Slominski A.T., Zmijewski M.A., Semak I., Zbytek B., Pisarchik A., Li W., Zjawiony J., Tuckey R.C. (2014). Cytochromes p450 and skin cancer: Role of local endocrine pathways. Anticancer Agents Med. Chem..

[50] Slominski A., Gomez-Sanchez C.E., Foecking M.F., Wortsman J. (1999). Metabolism of progesterone to DOC, corticosterone and 18OHDOC in cultured human melanoma cells. FEBS Lett..

[51] Camats N., Pandey A.V., Fernandez-Cancio M., Fernandez J.M., Ortega A.M., Udhane S., Andaluz P., Audi L., Fluck C.E. (2014). STAR splicing mutations cause the severe phenotype of lipoid congenital adrenal hyperplasia: Insights from a novel splice mutation and review of reported cases. Clin. Endocrinol..

[52] Miller W.L. (2007). Steroidogenic acute regulatory protein (StAR), a novel mitochondrial cholesterol transporter. Biochim. Biophys. Acta.

[53] Papadopoulos V., Aghazadeh Y., Fan J., Campioli E., Zirkin B., Midzak A. (2015). Translocator protein-mediated pharmacology of cholesterol transport and steroidogenesis. Mol. Cell. Endocrinol..

[54] Fan J., Wang K., Zirkin B., Papadopoulos V. (2018). CRISPR/Cas9Mediated Tspo Gene Mutations Lead to Reduced Mitochondrial Membrane Potential and Steroid Formation in MA-10 Mouse Tumor Leydig Cells. Endocrinology.

[55] Strauss J.F., Kishida T., Christenson LK., Fujimoto T., Hiroi H. (2003). START domain proteins and the intracellular trafficking of cholesterol in steroidogenic cells. Mol. Cell. Endocrinol..

[56] Alpy F., Legueux F., Bianchetti L., Tomasetto C. (2009). START domain-containing proteins: A review of their role in lipid transport and exchange. Med. Sci. Paris.

[57] Tomasetto C., Regnier C., Moog-Lutz C., Mattei M.G., Chenard M.P., Lidereau R., Basset P., Rio M.C. (1995). Identification of four novel human genes amplified and overexpressed in breast carcinoma and localized to the q11-q21.3 region of chromosome 17. Genomics.

[58] Akiyama N., Sasaki H., Ishizuka T., Kishi T., Sakamoto H., Onda M., Hirai H., Yazaki Y., Sugimura T., Terada M. (1997). Isolation of a candidate gene, CAB1, for cholesterol transport to mitochondria from the c-ERBB-2 amplicon by a modified cDNA selection method. Cancer Res..

[59] Alpy F., Boulay A., Moog-Lutz C., Andarawewa K.L., Degot S., Stoll I., Rio M.C., Tomasetto C. (2003). Metastatic lymph node 64 (MLN64), a gene overexpressed in breast cancers, is regulated by Sp/KLF transcription factors. Oncogene.

[60] Vassilev B., Sihto H., Li S., Holtta-Vuori M., Ilola J., Lundin J., Isola J., Kellokumpu-Lehtinen P.L., Joensuu H., Ikonen E. (2015). Elevated levels of StAR-related lipid transfer protein 3 alter cholesterol balance and adhesiveness of breast cancer cells: Potential mechanisms contributing to progression of HER2-positive breast cancers. Am. J. Pathol..

[61] Llaverias G., Danilo C., Mercier I., Daumer K., Capozza F., Williams T.M., Sotgia F., Lisanti M.P., Frank P.G. (2011). Role of cholesterol in the development and progression of breast cancer. Am. J. Pathol..

[62] Voisin M., de Medina P., Mallinger A., Dalenc F., Huc-Claustre E., Leignadier J., Serhan N., Soules R., Segala G., Mougel A. (2017). Identification of a tumor-promoter cholesterol metabolite in human breast cancers acting through the glucocorticoid receptor. Proc. Natl. Acad. Sci. USA.

[63] Simpson E., Santen R.J. (2015). Celebrating 75 years of oestradiol. J. Mol. Endocrinol..

[64] Zhao H., Zhou L., Shangguan A.J., Bulun S.E. (2016). Aromatase expression and regulation in breast and endometrial cancer. J. Mol. Endocrinol..

[65] Manna P.R., Chandrala S.P., King S.R., Jo Y., Counis R., Huhtaniemi I.T., Stocco D.M. (2006). Molecular mechanisms of insulin-like growth factor-I mediated regulation of the steroidogenic acute regulatory protein in mouse leydig cells. Mol. Endocrinol..

[66] Manna P.R., Soh J.W., Stocco D.M. (2011). The involvement of specific PKC isoenzymes in phorbol ester-mediated regulation of steroidogenic acute regulatory protein expression and steroid synthesis in mouse Leydig cells. Endocrinology.

[67] Arakane F., King S.R., Du Y., Kallen C.B., Walsh L.P., Watari H., Stocco D.M., Strauss J.F. (1997). Phosphorylation of steroidogenic acute regulatory protein (StAR) modulates its steroidogenic activity. J. Biol. Chem..

[68] Clark B.J., Ranganathan V., Combs R. (2001). Steroidogenic acute regulatory protein expression is dependent upon post-translational effects of cAMP-dependent protein kinase A. Mol. Cell. Endocrinol..

[69] Castro-Piedras I., Sharma M., den Bakker M., Molehin D., Martinez E.G., Vartak D., Pruitt W.M., Deitrick J., Almodovar S., Pruitt K. (2018). DVL1 and DVL3 differentially localize to CYP19A1 promoters and regulate aromatase mRNA in breast cancer cells. Oncotarget.

[70] Simpson E.R., Misso M., Hewitt K.N., Hill R.A., Boon W.C., Jones M.E., Kovacic A., Zhou J., Clyne C.D. (2005). Estrogen--the good, the bad, and the unexpected. Endocr. Rev..

[71] Molehin D., Castro-Piedras I., Sharma M., Sennoune S.R., Arena D., Manna P.R., Pruitt K. (2018). Aromatase Acetylation Patterns and Altered Activity in Response to Sirtuin Inhibition. Mol. Cancer Res..

[72] Bulun S.E., Lin Z., Imir G., Amin S., Demura M., Yilmaz B., Martin R., Utsunomiya H., Thung S., Gurates B. (2005). Regulation of aromatase expression in estrogen-responsive breast and uterine disease: From bench to treatment. Pharmacol. Rev..

