# Supplementary material for: Genomic Profiling of the Steroidogenic Acute Regulatory Protein in Breast Cancer: In Silico Assessments and a Mechanistic Perspective
Source: Cancers (Basel). 2019 May 4;11(5):623. doi: 10.3390/cancers11050623 (PMC6562549; doi:10.3390/cancers11050623)
Supplement: Supplementary file 1 [file cancers-11-00623-s001.zip › cancers-498220-supplement/cancers-498220-supplementary figures.pdf]

Supplemental materials: Genomic Profiling of the Steroidogenic Acute Regulatory Protein in Breast Cancer: In Silico Assessments and a Mechanistic Perspective

Pulak R. Manna, Ahsen U. Ahmed, Shengping Yang, Madhusudhanan Narasimhan, Joëlle Cohen-Tannoudji, Andrzej T. Slominski and Kevin Pruitt

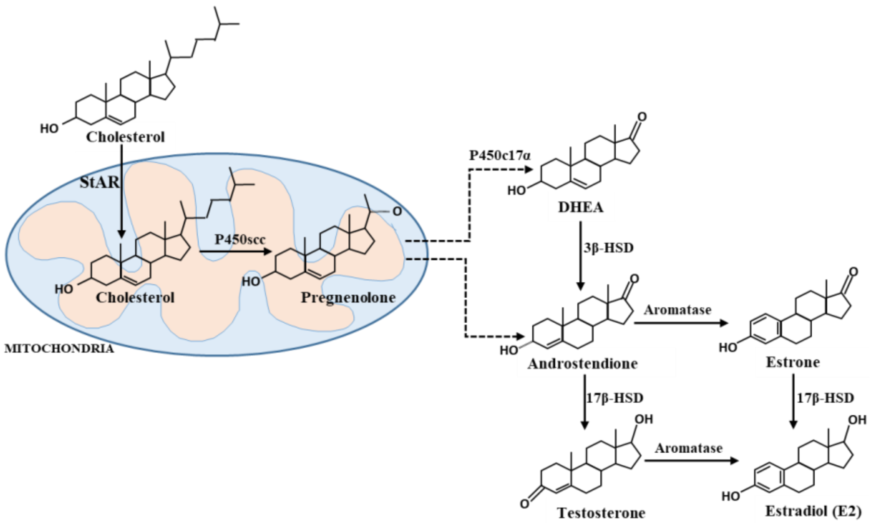

Figure S1. Steroid biosynthetic pathway. The StAR protein regulates the rate-limiting step in steroid biosynthesis i.e. the transport of cholesterol from the outer to the inner mitochondrial membrane, in which cholesterol is converted to pregnenolone by the P450scc enzyme (*CYP11A1*). Pregnenolone exits the mitochondria and then it is converted to various sex steroid hormones by a series of enzymes. StAR and key steroidogenic enzyme genes (for which proteins are illustrated by oval shapes), to E2 biosynthesis, were analyzed for their correlation to breast cancer. E2 induces breast cancer risk and progression. Shown are chemical structures of cholesterol and major steroid hormones.

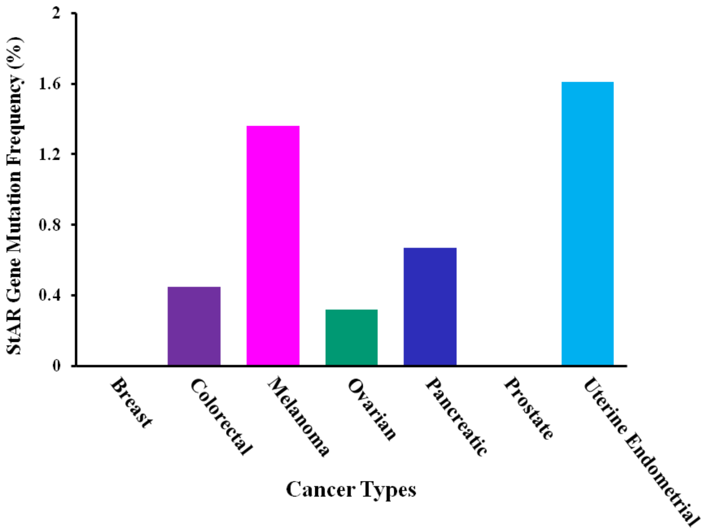

Figure S2. Assessment of mutation in the *StAR* gene in a variety of hormone sensitive cancers. Mutation in the *StAR* gene was assessed using whole exome sequencing with TCGA datasets for breast (982 tumors), colorectal (223 tumors), melanoma (368 tumors), ovarian (316 tumors), pancreatic

(150 tumors), prostate (499 tumors), and uterine endometrial (248 tumors) cancers. Note tumor numbers with complete/verified sequencing, different from Table 1, were evaluated for mutational analysis in the *StAR* gene.

© 2019 by the authors. Licensee MDPI, Basel, Switzerland. This article is an open access article distributed under the terms and conditions of the Creative Commons Attribution (CC BY) license (http://creativecommons.org/licenses/by/4.0/).
